# Spatial-Temporal Convolutional Transformer Network for Multivariate Time Series Forecasting

**DOI:** 10.3390/s22030841

**Published:** 2022-01-22

**Authors:** Lei Huang, Feng Mao, Kai Zhang, Zhiheng Li

**Affiliations:** 1 Tsinghua Shenzhen International Graduate School, Tsinghua University, Shenzhen 518055, China; huangl19@mails.tsinghua.edu.cn (L.H.); mf19@mails.tsinghua.edu.cn (F.M.); zhangkai@sz.tsinghua.edu.cn (K.Z.); 2 Department of Automation, Tsinghua University, Beijing 100086, China; 3 Research Institute of Tsinghua, Pearl River Delta, Guangzhou 510530, China

**Keywords:** multivariate time series forecasting, spatiotemporal, convolutional Transformer, attention mechanism

## Abstract

Multivariate time series forecasting has long been a research hotspot because of its wide range of application scenarios. However, the dynamics and multiple patterns of spatiotemporal dependencies make this problem challenging. Most existing methods suffer from two major shortcomings: (1) They ignore the local context semantics when modeling temporal dependencies. (2) They lack the ability to capture the spatial dependencies of multiple patterns. To tackle such issues, we propose a novel Transformer-based model for multivariate time series forecasting, called the spatial–temporal convolutional Transformer network (STCTN). STCTN mainly consists of two novel attention mechanisms to respectively model temporal and spatial dependencies. Local-range convolutional attention mechanism is proposed in STCTN to simultaneously focus on both global and local context temporal dependencies at the sequence level, which addresses the first shortcoming. Group-range convolutional attention mechanism is designed to model multiple spatial dependency patterns at graph level, as well as reduce the computation and memory complexity, which addresses the second shortcoming. Continuous positional encoding is proposed to link the historical observations and predicted future values in positional encoding, which also improves the forecasting performance. Extensive experiments on six real-world datasets show that the proposed STCTN outperforms the start-of-the-art methods and is more robust to nonsmooth time series data.

## 1. Introduction

Time series forecasting has a wide range of application scenarios in transportation, finance, medical, and other fields. Precise forecasting of time series can help people prepare for future changes, assist production management decisions, and demonstrate its important application value in traffic jam prevention, financial investment decisions, disease prevention, etc. [1,2,3].

The challenge of multivariate time series forecasting is the need to simultaneously capture complex spatiotemporal dependencies, which are mainly reflected in two aspects:

- Dynamic. Due to the changes in the external environment (such as events, weather, etc.), the spatiotemporal dependencies will dynamically change over time.
- Multiple patterns. Both temporal and spatial dependencies have multiple patterns. The temporal dependencies not only depend on the pointwise value of the observation point but also the local context of the surrounding observation points. In the spatial dimension, we need to consider not only local connectivity but also global semantic proximity. For example, in traffic time series, road nodes belonging to the same type of functional area have strong global semantic proximity, although they are not adjacent geographically [4,5].

Many achievements have been made in the study of time series in the past decades. Early time series forecasting studies mainly relied on statistical models, including autoregressive (AR), ARIMA [6], VAR [7], fuzzy method [8], etc. In order to extract nonlinear dependencies of time series, many machine learning and deep network methods were proposed, such as SVR [9], FC-LSTM [10], LSTNet [11], DBN [12], ST-ResNet [13], etc. The development of graph neural networks (GNNs) has brought time series forecasting to a new level and numerous GNN-based methods for spatiotemporal data prediction have been proposed, such as DCRNN [14], STGCN [15], ASTGCN [16], MTGNN [17], STSGCN [18], StemGNN [19], etc. Although these methods have shown good ability in time series forecasting tasks, they also face two major shortcomings.

First, basically all forecasting methods only consider the relationships between different time steps when modeling temporal dependency but do not consider the dependencies between time periods. External events often occur within a period and will affect the values of multiple consecutive observation points in the time series, and this is a local context semantic. The local context semantics should also be an important consideration when modeling temporal dependencies.

Second, from the perspective of spatial dependencies, the univariate time series forecasting methods [6,7,10,11,20,21] does not consider the spatial dependencies between multiple variables in the time series, and some of the latest deep learning methods either rely on a predefined graph structure or only learn a stable spatial relationship among multiple time series [4,14,15,16,17], which limits their ability to capture spatial dependencies with multiple patterns.

In this work, we innovate the Transformer framework to tackle these two shortcomings and present a novel Transformer-based method named spatial–temporal convolutional Transformer network (STCTN) for multivariate time series forecasting tasks. In STCTN, we proposed two novel attention mechanisms, namely the local-range convolutional attention mechanism and the group-range convolutional attention mechanism, which address the two shortcomings we have aforementioned. The two novel attention mechanisms capture multiple patterns of temporal dependencies and spatial dependencies at the sequence level and graph level, respectively. The local-range convolutional attention mechanism exploits convolutional kernels with various sizes to learn rich local context and simultaneously focus on both global and local context dependencies, which addresses the first shortcoming. The group-range convolutional attention mechanism uses multihead attention to learn the latent graph structures among multiple time series, extracting dynamic and multimodal spatial dependencies, which addresses the second shortcoming. In summary, our main contributions are as follows:

- We design a novel Transformer-based encoder–decoder framework for multivariate time series forecasting that can dynamically model spatiotemporal dependencies.
- Two novel range convolutional attention mechanisms are proposed to effectively extract dynamic and multimodal spatiotemporal dependencies and also reduce the computation complexity.
- Continuous positional encoding is also proposed to link the historical observations and predicted future values in positional encoding and improve prediction performance.

The rest of this paper is organized as follows. In Section 2, we briefly review the existing time series forecasting approaches. In Section 3, we define the multivariate time series forecasting problem and introduces the self-attention mechanism in Transformer as the background of this work. In Section 4, we describe in detail the proposed framework and elaborates the components. In Section 5, we conduct extensive experiments in six real-world datasets and compare the proposed method with ten baselines. We also conduct ablation experiments and model analysis in Section 5. This paper ends with conclusions and the future work in Section 6.

## 2. Related Work

Time series forecasting has been an important topic in data mining for decades. Early time series forecasting studies mainly relied on statistical models, including historic average (HA), autoregressive (AR), autoregression integrated moving average (ARIMA) [6], VAR [7], fuzzy method [8], etc. These statistical models have strong stationary assumptions and are difficult to capture nonlinear dependencies in time series. Machine learning and neural network methods can capture the nonlinear characteristics of time series more effectively. The RNN-based methods [22,23,24,25] adopt the recurrent neural network or its variants to capture nonlinear time patterns. Yu et al. [26] utilized matrix factorization to model the relationship across multiple time series. However, these models either ignore the spatial dependencies among multiple variables or only capture linear spatial dependencies, making them perform poorly in actual predictions.

Spatial–temporal forecasting methods hope to capture both temporal and spatial dependencies. Convolutional neural networks (CNNs) have shown powerful performance in learning local and shift-variant features [27]. There are some methods to model the spatial features using CNNs [13,22,28,29,30,31]. Lv et al. [30] integrated an RNN and CNN, whereby the RNN was in charge of temporal features and used CNN to capture the spatial features. Ma et al. [31] adopted deep CNNs for traffic speed prediction. Zhang et al. [13] proposed ST-ResNet based on residual convolution network for crowd flows prediction. However, these methods can only be used for standard grid data. ConvLSTM [22] extended the convolutional network to long short-term memory (LSTM) network to extract spatial and temporal information separately. Graph neural networks have achieved great success in capturing the spatial dependency of unstructured data [14,15,16,17,18,19,32,33,34]. DCRNN and STGCN [14,15] are the first two studies that introduced graph convolution networks into spatial–temporal data forecasting to better model spatial dependency. ASTGCN [16] added attention layers to the spatiotemporal network to capture the dynamics of spatiotemporal dependencies. Some methods [4,17,18,19] adaptively learn the adjacency matrix to solve the limitation that the general GNN-based methods require a predefined graph. However, these methods only learn a stable graph adjacency matrix, which limits their ability to capture spatial dependencies with multiple patterns.

Transformer [35] is a novel sequence modeling architecture, which introduced the self-attention mechanism to learn long-range sequential dependency. Transformer has achieved great success in many fields [35,36,37,38,39,40,41,42]. In particular, for time series forecasting tasks, Li et al. [38] proposed a method to enhance the locality and break the memory bottleneck of Transformer on time series forecasting. Wu et al. [39] proposed a new time series forecasting model named adversarial sparse Transformer based on generative adversarial networks. The obstacles of applying Transformer to multivariate time series forecasting are that the standard self-attention mechanism is only used at the sequence level and cannot capture the spatial dependencies, and it is also weak in capturing the temporal dependencies of multiple patterns. How to solve the challenges and extract the complex spatiotemporal dependencies are still the key issues in extending the Transformer to multivariate time series forecasting.

## 3. Background

### 3.1. Problem Definition

Let $X=\{x_{t}^{i}\}\in {\mathbb{R}}^{N\times T}$ denote N related univariate time series, where *T* is the number of timestamps and $x_{t}^{i}\in \mathbb{R}$ denotes the value of time series i at time *t*. We denote the observation values of multivariate time series at time t as $X_{t}=\{x_{t}^{1},x_{t}^{2},\cdots ,x_{t}^{N}\}$. The multivariate time series forecasting problem can be described as: learning a mapping function f that maps the observed values of historic *P* time steps $\left[X_{t-P+1},X_{t-P},\cdots ,X_{t}\right]$ into the future values of next Q time steps $\left[X_{t+1},X_{t+2},\cdots ,X_{t+Q}\right]$.

(1) $$\left[X_{t-P+1:t}]\overset{f}{\to }[X_{t+1:t+Q}\right]$$

### 3.2. Self-Attention Mechanism

The self-attention transforms the input $Z\in {\mathbb{R}}^{n\times d}$ into query matric $Q=ZW^{Q}$, key matric $K=ZW^{K}$, and value matric $V=ZW^{V}$, where $W^{Q},W^{K}\in {\mathbb{R}}^{d\times d_{k}}$ and $W^{V}\in {\mathbb{R}}^{d\times d_{v}}$ are learnable parameters. After those linear projections, the scaled dot-product attention computes the attention scores α:

(2) $$\alpha =\mathrm{softmax}(\frac{QK^{T}}{\sqrt{d_{k}}})\;or\;\alpha =\mathrm{softmax}(\frac{QK^{T}}{\sqrt{d_{k}}}\cdot M)$$

where M is the mask matrix with all upper triangular elements set to −∞. When the mask option is chosen, the mask matrix is applied to filter out rightward attention. Afterward, the output of the self-attention is:

(3) O=Attention(Q,K,V)=αV

## 4. Methodology

### 4.1. The Overall Architecture

**Figure 1:**
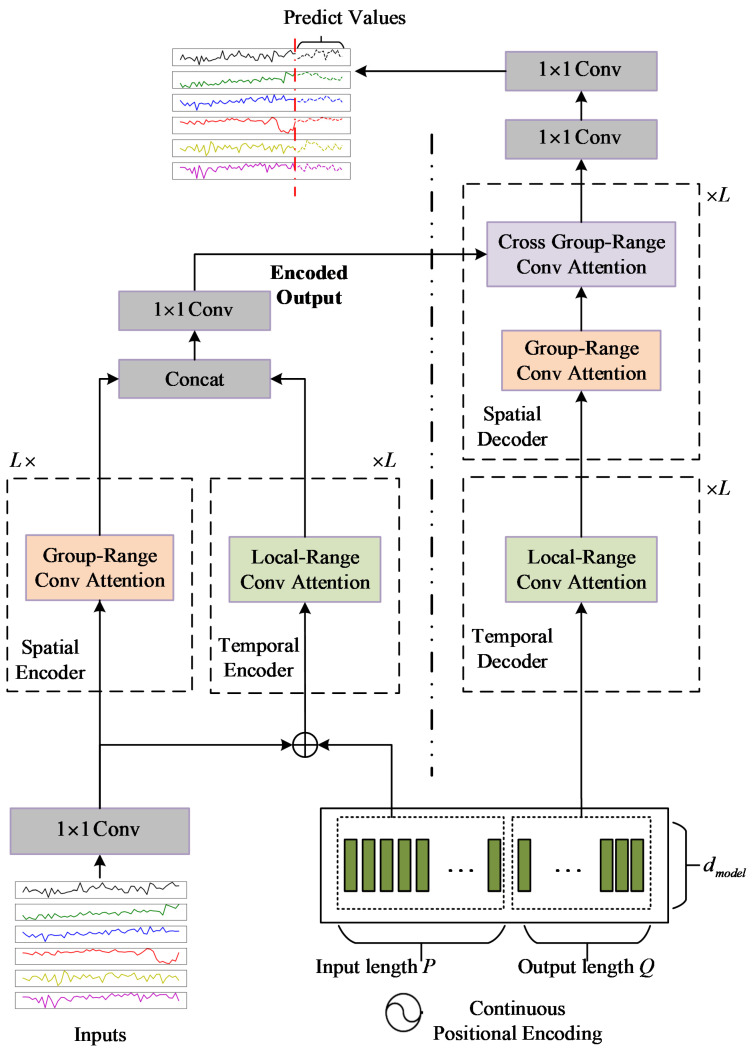
The framework of STCTN. “⊕” denotes the elementwise addition. For simplicity, we hide all the residual connections, layer regularizations, and fully connected feed-forward networks that are similar to the standard Transformer [35].

Many competitive neural networks for time series modeling have an encoder–decoder structure [14,39,43]. Here, the STCTN also follows a Transformer encoder–decoder structure with multiattentions. Figure 1 illustrates the framework of our proposed STCTN, which consists of four components: continuous position encoding module, spatial–temporal encoder, spatial–temporal decoder, and output module. The spatial–temporal encoder is composed of a temporal encoder and a spatial encoder in parallel, which can facilitate the parallelization of model computing. The spatial–temporal decoder consists of a temporal decoder and a spatial decoder stacked successively. Both the encoders and the decoders are composed of a stack of L identical layers with residual connections. The outputs of the temporal encoder and the spatial encoder are fused to obtain an encoded output. Then, the outputs of the encoder are fed into the decoder to generate multistep predictions as a whole. Two kinds of novel attention mechanisms, that is, local-range convolutional attention and group-range convolutional attention, are also proposed in STCTN to improve the accuracy and efficiency of the network. To facilitate the residual connections, all layers in STCTN produce outputs of dimension $d_{model}$.

### 4.2. Novel Attention Mechanisms

In this section, we introduced in detail the novel attention mechanisms proposed in STCTN.

#### 4.2.1. Local-Range Convolutional Attention

The temporal dependency of multivariable time series has many patterns. External events often occur within a period and will affect the values of multiple consecutive observation points. However, the standard self-attention calculates the attention score through the pointwise value of the observation point, that is, it only pays attention to the dependency between points in the global scope, and ignores the relationship between different local contexts. In order to model these characteristics, we propose the local-range convolutional attention to simultaneously focus on the global dependency and local context dependency.

**Figure 2:**
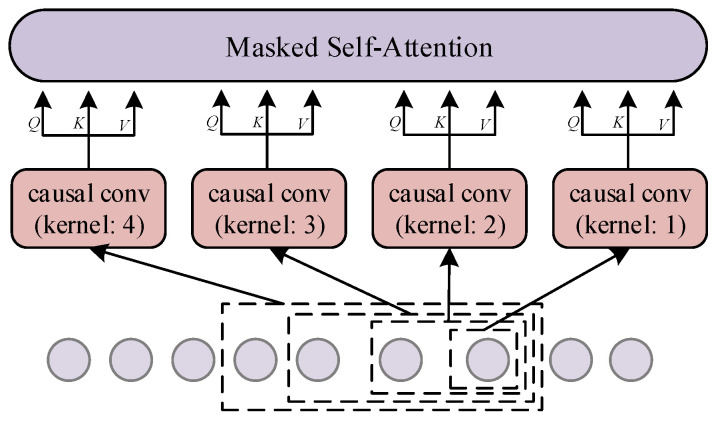
The Local-Range Convolutional Attention. Q,K,V represent query matrix, key matrix, value matrix, respectively.

Local-range convolutional attention is applied at the sequence level and captures temporal multimodal dependencies, and its architecture is shown in Figure 2. Given the input to the local-range convolutional attention is $H_{T}\in {\mathbb{R}}^{N\times T\times d_{model}}$, we use causal convolution with S different kernel sizes to learn local context with different range sizes, and then use self-attention to learn temporal dependencies on local context representations. Unlike standard self-attention [35], we use causal convolution mapping instead of linear mapping to calculate query matrix, key matrix, and value matrix:

(4) $$\begin{array}{l} Q_{m}=f_{LRC}^{\left[m\right]}(H_{T})W_{m}^{Q}\\ K_{m}=f_{LRC}^{\left[m\right]}(H_{T})W_{m}^{K}\\ V_{m}=f_{LRC}^{\left[m\right]}(H_{T})W_{m}^{V} \end{array}$$

(5) $$Q_{LRC}^{m}=\alpha_{m}V_{m}=\mathrm{softmax}(\frac{Q_{m}K_{m}^{T}}{\sqrt{d_{k}}}\cdot M)V_{m}$$

where $f_{LRC}^{\left[m\right]}$ represents the causal convolution of kernel size m with stride 1 and proper paddings, $W_{m}^{Q},W_{m}^{K}\in {\mathbb{R}}^{d_{model}\times d_{k}}$ and $W_{m}^{V}\in {\mathbb{R}}^{d_{model}\times d_{v}}$ are learnable parameters. The parameters are shared among all variables. The final output of the local-range convolutional attention $O_{LRC}\in {\mathbb{R}}^{N\times T\times d_{model}}$ is the linear projection of the concatenation of $O_{LRC}^{1},O_{LRC}^{2},\cdots ,O_{LRC}^{S}$. In this paper, the size of the convolution kernels is {1,2,3,4}, respectively. When the kernel size is relatively large, a wide range of local contexts can be extracted. When the kernel size is 1, it just performs a linear mapping before computing the attention score, so that the model can still learn the dependency based on a pointwise value.

#### 4.2.2. Group-Range Convolutional Attention

**Figure 3:**
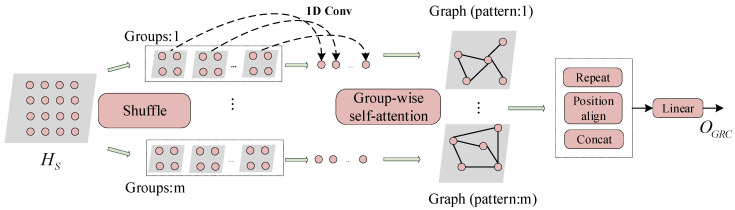
The Group-Range Convolutional Attention.

We use multihead attention to capture the latent relationships among multiple time series in different subspaces, that is, the latent graph structures. However, the standard self-attention has a time and memory complexity of $O(n^{2})$. On the other hand, since there is no or weak relationship among many variables in the time series, it is unnecessary to calculate the pairwise relationship between all nodes. To address those limitations, we propose the group-range convolutional attention which divides variable nodes into groups and calculates the groupwise relationships. Group-range convolutional attention is applied on graph level and the architecture is illustrated in Figure 3.

We employ 1D convolution of kernel size k with stride k to transform the inputs $H_{S}\in {\mathbb{R}}^{N_{g}\times T\times d_{model}}$ (with proper padding) into different groups. The number of groups is:

(6) $$N_{g}=[N/k]+1$$

where [⋅] represents the integer function. The convolution kernel k determines the group size and the number of groups. The 1D convolution gathers the node information within the group, and then the attention mechanism is applied to calculate the groupwise attention score matrix, which is served as the adjacency weight matrix for the graph. According to Equations (2) and (3), the outputs of groupwise attention are obtained as $O_{att}\in {\mathbb{R}}^{N_{g}\times T\times d_{model}}$.

##### Shuffle Operation

To extract the spatial dependencies of multiple patterns, we need to perform 1D convolution and groupwise self-attention many times. However, there is a problem that 1D convolution is carried out according to the row order of the input matrix, if we do not change the row order, the grouping results are the same. Since the node variables do not have order relationships in the real physical space, we do not need to consider the row order of the input matrix. Therefore, different groupings can be obtained by disrupting the order of node variables in the input matrix, the operation can be written as follows:

(7) $$H_{sf}^{i}=\mathrm{shuffle}(H_{S},0)\;i=1,2,\cdots ,h$$

where $\mathrm{shuffle}(H_{S},0)$ represents randomly permute the rows of the input matrix, and h denotes the grouping times.

##### Repeat Operation

To facilitate the residual connection behind the attentions, we use the repeat operation to convert the output of groupwise self-attention from $O_{att}^{i}\in {\mathbb{R}}^{N_{g}\times T\times d_{model}}$ to $O_{rp}^{i}\in {\mathbb{R}}^{N\times T\times d_{model}}$; the repeat operation can be represented as:

(8) $$\begin{array}{l} O_{att}^{i}=\{O_{1}^{i};\cdots ;O_{S}^{i};\cdots ;O_{N_{g}}^{i}\}\overset{repeat}{\to }O_{rp}^{i}\\ \;\;\;\;\;\;\;=\{O_{1}^{i};\cdots ;O_{1}^{i};\cdots ;O_{S}^{i};\cdots ;O_{S}^{i};\cdots O_{N_{g}}^{i};\cdots O_{N_{g}}^{i}\} \end{array}$$

(9) $$O_{rp}^{i}=O_{rp}^{i}[0:N,:,:]\;if\;(N_{g}\times k)>N$$

where $O_{S}^{i}$ represents row S of attention output matrix $O_{att}^{i}$. Here, we copy each row k times. Since the paddings in convolution may cause $(N_{g}\times k)>N$, we only take the previous N rows of $O_{rp}^{i}\in {\mathbb{R}}^{(N_{g}\times k)\times T\times d_{model}}$ from the repeat operation.

##### Position-Align Operation

The shuffle operation changes the original order of variable nodes. In order to concatenate the output of all groupwise self-attentions, we must restore the arrangement order of variable nodes in the repeat operation output $O_{repeat}^{i}$ to the arrangement order in the input $H_{S}$, which is the position-align operation. The position-align operation generates the output $O_{ali}^{i}\in {\mathbb{R}}^{N\times T\times d_{model}}$.

As depicted in Figure 3, after the shuffle operation, the group-range convolutional attention can learn different spatial dependency graph patterns in parallel; then, a series of outputs $O_{ali}^{1},O_{ali}^{2},\cdots ,O_{ali}^{h}$ are obtained after repeat operation and position-align operation. Finally, these outputs are concatenated and a linear mapping is performed to generate the final outputs $O_{GRC}\in {\mathbb{R}}^{N\times T\times d_{model}}$. The time and memory complexity are reduced by $k^{2}$ times through groupwise attention. This cuts of the bottleneck of computation and memory, and the increase in complexity caused by the convolution is acceptable compared to the gains obtained by the groupwise attention. The group-range convolutional attention algorithm is given in Algorithm 1.
**Algorithm 1:** Group-Range Convolutional Attention Algorithm**Input:** Node feature representations $H_{in}$, Number of nodes N, grouping times *m*, group size k**Output:** Learned spatial features $H_{out}$1:     compute the number of groups $N_{g}$2:     **for**
i=1 **to**
m **do**3:              $H_{sf}^{i}=\mathrm{ShuffleOperation}(H_{in})$4:              $H_{g}^{i}$ = Grouping $H_{sf}^{i}$ for $N_{g}$ groups with 1D convolution5:              $H_{att}^{i}=\mathrm{Attention}(W_{i}^{Q}H_{g}^{i},W_{i}^{K}H_{g}^{i},W_{i}^{V}H_{g}^{i})$6:              $O_{rp}^{i}=\mathrm{RepeatOperation}(H_{att}^{i})$7:              **if** $(N_{g}\times k)>N$ **then**8:                  $O_{rp}^{i}=O_{rp}^{i}[0:N]$9:              **end if**10:              $O_{ali}^{i}=\mathrm{PositionAlignOperation}(O_{rp}^{i})$11:     **end for**12:     $H_{out}=\mathrm{Linear}(\mathrm{Concat}[O_{ali}^{1},O_{ali}^{2},\cdots ,O_{ali}^{m}])$13:     **return**
$H_{out}$

### 4.3. Continuous Positional Encoding

Since our model contains no recurrence, to utilize the order of the sequence, we follow [35] to compute the positional encoding using sine and cosine functions of different frequencies:

(10) $$\begin{array}{l} PE_{\left(pos,2i\right)}=\sin(pos/10,000^{2i/d_{model}})\\ PE_{\left(pos,2i+1\right)}=\cos(pos/10,000^{2i/d_{model}}) \end{array}$$

where pos is the position and i is the dimension. However, almost all Transformer frameworks that have position encoding [35,44] use independent position encoding for the encoder and decoder. In time series forecasting tasks, the historical observations and predicted future values are not independent in sequence and have a front-to-back position relationship. To consider this position relationship, we design continuous positional encoding, as shown at the bottom of Figure 1. The positioned embeddings are calculated as follows:

(11) $$PE=\{PE_{0},PE_{1},\cdots ,PE_{P+Q}\}$$

(12) $$X_{ENC}^{in}=X^{in}⊕PE_{1:P}$$

(13) $$X_{DEC}^{in}=PE_{P+1:P+Q}$$

where $PE_{1:P}\in {\mathbb{R}}^{N\times P\times d_{model}}$ denote the first P columns and $PE_{\left(P+1:P+Q\right)}\in {\mathbb{R}}^{N\times Q\times d_{model}}$ denotes the last Q columns of the second dimension of *PE*, the ⊕ operation represents the elementwise addition. $X_{ENC}^{in}$ is then used as input to the encoder and $X_{DEC}^{in}$ is used as input to the decoder.

### 4.4. Spatial–Temporal Encoder

As shown in Figure 1, the encoder is composed of a spatial encoder and a temporal encoder in parallel. Each spatial encoder layer contains two sublayers, which are group-range convolutional attention and fully connected feed-forward network. We also employ the residual connection and layer normalization around each of the sublayer similar to the standard Transformer [35]. The historical observation data is first transformed to $H_{PE}^{in}$ using 1×1 convolution layer and then fed into the spatial encoder to produce the output $H_{SE}^{out}\in {\mathbb{R}}^{N\times P\times d_{model}}$. Each temporal encoder layer consists of a local-range convolutional attention mechanism and a feed-forward network. The other parts are the same as the spatial encoder. After continuous position embedding, $X_{ENC}^{in}$ are fed to the temporal encoder and produce output $H_{TE}^{out}\in {\mathbb{R}}^{N\times P\times d_{model}}$.

The spatial encoder and the temporal encoder respectively generate outputs $H_{SE}^{out}$ and $H_{TE}^{out}$. These outputs are concatenated and then a 1×1 convolution layer is used to generate the final output of the encoder module $H_{ENC}^{out}\in {\mathbb{R}}^{N\times P\times d_{model}}$.

### 4.5. Spatial–Temporal Decoder

The decoder is composed of a temporal decoder and a spatial decoder serially. Each temporal decoder layer uses the local-range attention mechanism. The temporal decoder takes the output of the continuous positional encoding module $X_{DEC}^{in}$ as input and generates output $H_{TD}^{out}\in {\mathbb{R}}^{N\times Q\times d_{model}}$ after L stacked layers. The attention mechanism used in the spatial decoder layer is group-range convolutional attention. The spatial decoder has another sublayer, the cross group-range convolutional attention, which performs attention over the encoded output $H_{ENC}^{out}$. The spatial decoder generates the final output of the spatial–temporal decoder $H_{DEC}^{out}\in {\mathbb{R}}^{N\times Q\times d_{model}}$.

### 4.6. Output Module

In the output module, the spatial–temporal features output by the final spatial decoder are fed as input. The output module consists of two 1×1 standard convolution layers, transforming the final decoded output $H_{DEC}^{out}$ into the expected prediction output $\hat{Y}\in {\mathbb{R}}^{N\times Q}$, which can be formulated as:

(14) $$\hat{Y}=\mathrm{Conv}(\mathrm{Conv}(H_{DEC}^{out}))$$

Mean absolute error (MAE) between predicted values and ground truths are then adopted to train the model as:

(15) $$Loss=\frac{1}{N\times T}\sum_{i=1}^{N}\sum_{t=P+1}^{P+Q}\left|Y_{t}^{i}-{\hat{Y}}_{t}^{i}\right|$$

## 5. Experiments

### 5.1. Datasets and Data Preprocessing

We evaluate the performance of STCTN on six public datasets. The PEMS03, PEMS04, PEMS07, and PEMS08 are traffic time series datasets with priori graph topology, released by [45]. Traffic and Electricity are pure multivariate time series datasets without priori graph topology, released by [11].

PEMS03, PEMS04, PEMS07, and PEMS08 are collected by the Caltrans Performance Measurement System (PEMS). The four datasets are constructed from four different districts in California and aggregated into 5 min from the raw data which was sampled every 30 s. Each dataset records three different road attributes: traffic flow, average speed, and average occupancy. We evaluate the performance of traffic flow forecasting in our experiments. In particular, the distance between the sensors recorded in the dataset was used to construct the prior graph topology.

Traffic, Electricity are pure multivariate times series datasets without prior graph topology. The Traffic dataset describes the road occupancy rates measured by 862 sensors in San Francisco Bay area freeways. The Electricity dataset recorded the electricity consumption of 321 clients from 2012 to 2014. The sampling interval of both datasets is one hour. In particular, following [46], the electricity data is first transformed into a range of 0 to 1 and the evaluation is performed on the rescaled data for all the methods.

For the PEMS datasets, we use one-hour historical data to predict the next hour data and evaluate the average prediction result. Therefore, the input sequence length and the output sequence length are both 12. For the Traffic and Electricity datasets, we use 24-h historic data to predict the values in the next 12 h, and evaluate the prediction results of step 3, step 6, and step 12. The input sequence length is 24 and the output sequence is 12. All the inputs are normalized by the Z-Score method as $X^{norm}=(X-\mu (x))/\sigma (X)$, where μ denotes the mean value and σ denotes the standard deviation. The dataset description and statistics are summarized in Table 1.

### 5.2. Baseline Methods

To assess the performance of our method, we compare STCTN with the traditional time series analysis method VAR [7], the latest deep learning methods including FC-LSTM [10], N-BEATS [47], Transformer-based models (i.e., Transformer [35], informer [43]), and several GNN-based models (i.e., DCRNN [14], STGCN [15], ASTGCN [16], Graph Wavenet [4], MTGNN [17]). Note that other GNN-based methods except Graph WaveNet and MTGNN require a predefined graph, and they can only be used in the datasets with a priori graph topology. The detail of the baselines are as follows:

- VAR: An advanced time series model, which can capture the pairwise relationships among time series [7].
- FC-LSTM: A recurrent neural network with fully connected LSTM hidden units [10].
- DCRNN: Diffusion convolutional recurrent neural network that integrates graph convolution into sequence-to-sequence architecture [14].
- STGCN: Spatial–temporal graph convolutional network, which integrates graph convolution into 1D convolution [15].
- ASTGCN: Attention-based spatial–temporal graph convolutional network, which designs temporal and spatial attention mechanisms [16].
- Graph WaveNet: A spatial–temporal graph convolutional network, which combines graph convolution with dilated causal convolution [4].
- MTGNN: Multivariate time series forecasting model with graph neural networks, which utilizes a graph learning module to extract the relations among variables [17].
- N-BEATS: A deep learning architecture based on backward and forward residual links and fully connected layers [47].
- Transformer: The first deep learning network that proposed a self-attention mechanism and used it for sequence modeling tasks [35].
- Informer: A deep learning method based on Transformer, which improves the attention mechanism for long series time series prediction [43].

### 5.3. Experimental Settings

All the datasets are split into training sets, validation sets, and test sets with a ratio of 6:2:2. To prevent information leakage in the future, we keep the chronological order of the data when splitting the dataset, that is, the sampling time of the training data is always before the test data. We also use the time of the day as an auxiliary feature.

All the experiments are conducted under the environment with one Inter(R) Xeon(R) CPU E5-4650 V4 @ 2.20GHz and two NVIDIA TITAN RTX GPU cards. Adam optimizer is chosen to train our model with gradient clip 5. The initial learning rate is 0.001 and the weight decay is 0.0001. We train the model 100 epochs on each dataset and the batch size is 32. Dropout with 0.3 is applied after each stacked layer of encoder and decoder. Early stop strategy was applied during the training process to prevent overfitting. The performance of the model on the validation set is evaluated at the end of each epoch. When the loss on the validation set does not decrease for 20 consecutive epochs, the training is stopped. We save the model that performs best on the validation set during training and used it for testing. Other hyperparameters vary by dataset.

- PEMS08. The number of model channels $d_{model}$ is 16. The number of stacked layers is set to 4 and the group size in the group-range convolutional attention is set to 10.
- PEMS03, PEMS04, and Electricity. The number of model channels is 16. The number of stacked layers is set to 4 and the group size is set to 50.
- PEMS07 and Traffic. The number of model channels is 8. The number of stacked layers is set to 3 and the group size is set to 100.

### 5.4. Evaluation Metrics

Following [17], we use three widely used metrics to evaluate the performance, including mean absolute error (MAE), mean absolute percentage error (MAPE), and root mean square error (RMSE). Lower values mean better performance for those metrics. MAE, MAPE, and RMSE can be calculated as follows:

(16) $$MAE=\frac{1}{N\times T}\sum_{i=1}^{N}\sum_{t=1}^{T}\left|Y_{t}^{i}-{\hat{Y}}_{t}^{i}\right|$$

(17) $$MAPE=\frac{1}{N\times T}\sum_{i=1}^{N}\sum_{t=1}^{T}\frac{\left|Y_{t}^{i}-{\hat{Y}}_{t}^{i}\right|}{Y_{t}^{i}}\times 100\%$$

(18) $$RMSE=\sqrt{\frac{1}{N\times T}\sum_{i=1}^{N}\sum_{t=1}^{T}{\left(Y_{t}^{i}-{\hat{Y}}_{t}^{i}\right)}^{2}}$$

where $Y_{t}^{i}$ and ${\hat{Y}}_{t}^{i}$ are predicted values and ground truths of the $i^{\mathrm{th}}$ time series at time step t, respectively.

### 5.5. Results and Analysis

The main experimental results are shown in Table 2 and Table 3. Table 2 shows the comparison of the average prediction performance of multistep (12 steps) predictions on datasets with a priori graph topology. Table 3 shows the comparison of different approaches for 3-step, 6-step, and 12-step ahead predictions on datasets without priori graph topology.

Table 2 shows that our STCTN achieves the start-of-the-art prediction performance in almost all the datasets and metrics. In the baseline models, DCRNN, STGCN, and ASTGCN capture spatial dependencies based on a predefined graph, they perform better than methods that only consider temporal dependencies. However, this limits their application to pure multivariate time series without a predefined graph. Graph WaveNet and MTGNN develop an adaptive dependency matrix to represent the spatial correlations, but the dependency matrix is fixed once learned without considering the dynamics and multipatterns of the spatial dependencies. Compared to the GNN-based models that rely on a predefined graph or learn fixed graph structure from data, STCTN still achieves state-of-the-art prediction performance without the aid of a predefined graph.

For the datasets without prior graph topology in Table 3, STCTN significantly outperforms the baseline methods in all steps. Compared to Transformer and Informer, our proposed method still achieves the best performance. The advantage of Informer lies in the long sequence time series prediction, and it does not consider the spatial dependencies between multiple variables, so STCTN outperforms Informer on the relatively short-term prediction tasks. In particular, in the Traffic dataset that is not so smooth, the performance of Graph WaveNet decreases significantly, indicating that it is more suitable for modeling smooth data, and our method is robust to both smooth and unsmooth time series data.

**Figure 4:**
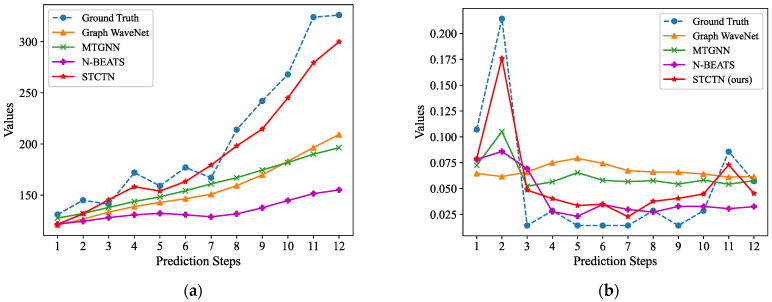
The forecasting results of 12 steps on different datasets. (**a**) PEMS08; (**b**) Electricity.

**Figure 5:**
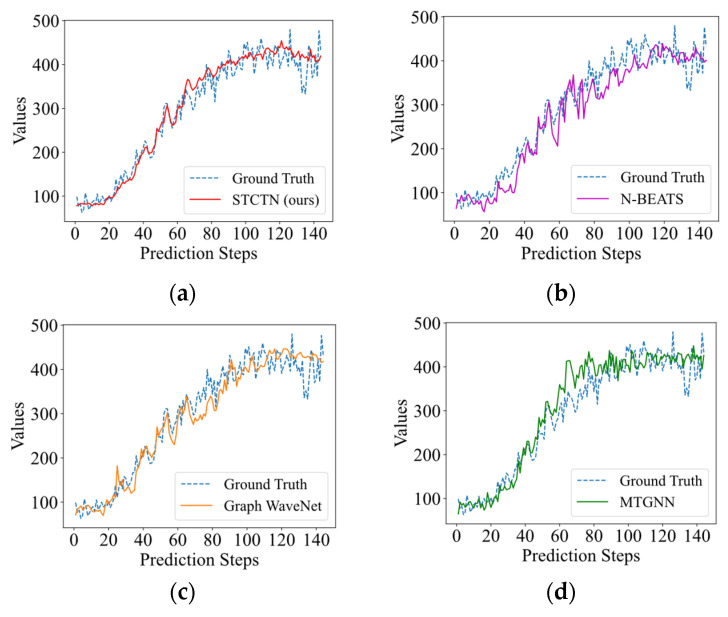
The long-term forecasting results (12 h, 144 steps). (**a**) STCTN (ours); (**b**) N-BEATS; (**c**) Graph WaveNet; (**d**) MTGNN.

**Figure 6:**
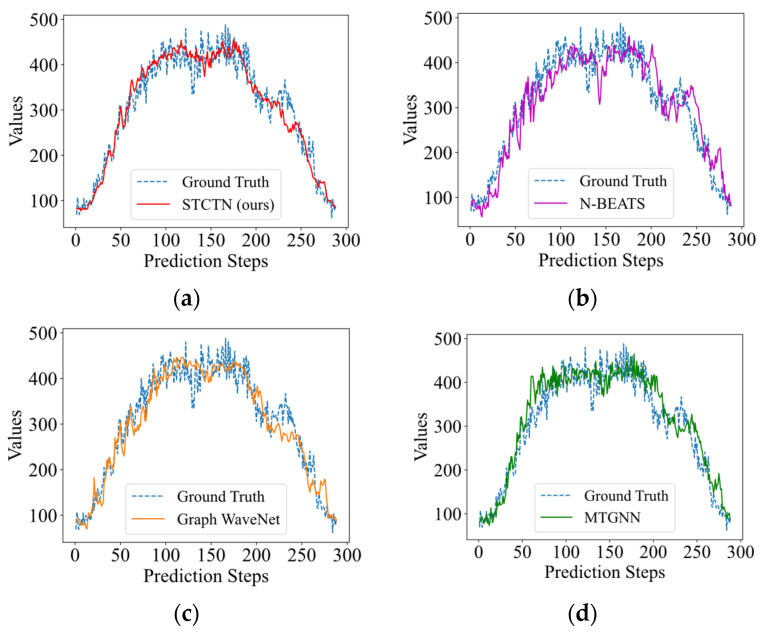
The long-term forecasting results (one day, 288 steps). (**a**) STCTN (ours); (**b**) N-BEATS; (**c**) Graph WaveNet; (**d**) MTGNN.

The multistep (12 steps) forecasting results on the PEMS08 and Electricity datasets are shown in Figure 4. It shows that the multistep outputs generated by the baseline models are relatively smooth, they always fit the overall trends while ignoring most of the fluctuation information. Our method can pay more attention to the fluctuations and fit the fluctuations more effectively. We further illustrate the visualization prediction results with long-term prediction (144 steps and 288 steps) for baselines and STCGN, which are shown in Figure 5 and Figure 6. As we can observe that compared with N-Beats, Graph WaveNet, and MTGNN, our model more accurately follows the changes of ground truth.

### 5.6. Ablation Study

To better understand the effectiveness of the key components of STCTN, we design three variants of STCTN and conduct ablation experiments. The differences of the variants are described as follows:

- w/o CPE: We use the independent position encoding in the encoder and decoder instead of continuous position encoding.
- w/o LCA: We replace the local-range convolutional attention mechanism in both the encoder and decoder module with the standard multihead attention mechanism.
- w/o GCA: We replace the group-range convolutional attention mechanism in both the encoder and decoder module with the standard multihead attention mechanism.

**Figure 7:**
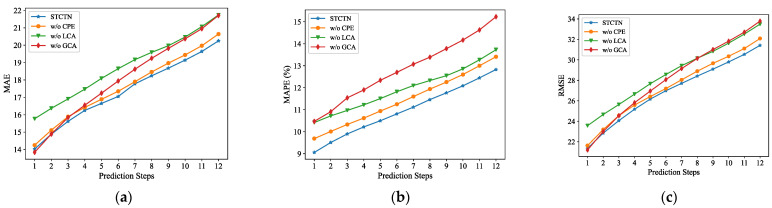
Errors in each prediction step of STCTN and the three variants. (**a**) MAE; (**b**) MAPE; (**c**) RMSE.

Table 4 represents the results obtained on the PMES08 dataset. It shows that these key components in STCTN are indispensable. As we replace the local-range convolutional attention mechanism with the standard multihead attention mechanism, the evaluation metrics increase by a great amount, which indicates that local context semantic is the important content of temporal dependencies. The group-range convolutional attention mechanism is proved to be effective because it dynamically models the spatial dependencies of multiple patterns. The continuous position encoding also helps improve forecasting performance. Figure 7 shows the MAE, MAPE, and RMSE in each prediction step of STCTN and the three variants. We observe that STCTN outperforms the variants in all prediction steps, indicating the effectiveness of the designed modules. Moreover, as the prediction step increases, the performance difference between the variants and STGCN gradually increases, indicating that STGCN has stronger long-term prediction capabilities.

### 5.7. Model Parameter Analysis

**Figure 8:**
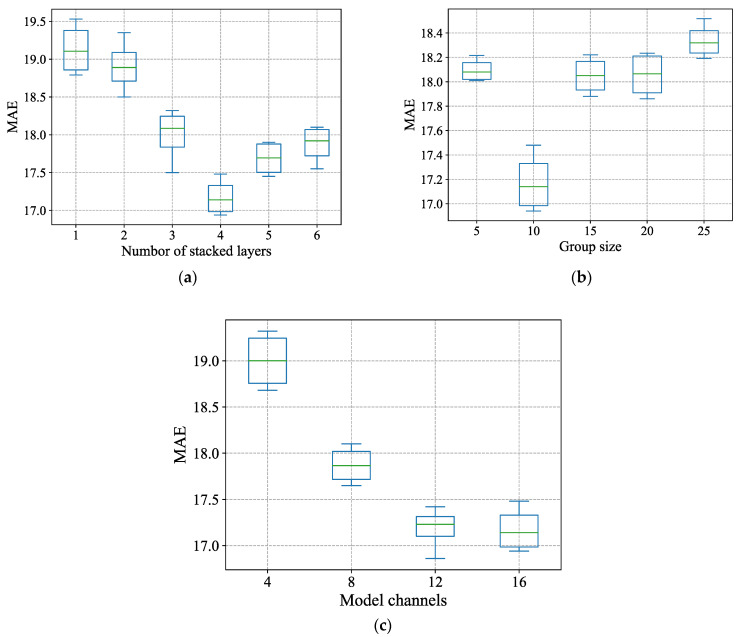
Parameter study. (**a**) Stacked layers; (**b**) group size; (**c**) model channels.

To assess the effect of hyperparameters on the model, we conducted a parameter study of three core parameters including the number of stacked layers L, the model channels $d_{model}$, and the range size of the group-range convolutional attention k. We conduct five experiments each time with other parameters fixed and report the average of MAE. The number of stacked layers ranges from 1 to 6. The range size of group-range convolution attention ranges from 5 to 25. The number of model channels ranges from 4 to 16, with step size of 4. All the experiments are conducted on the PEMS08 dataset. The boxplot of the results is shown in Figure 8. STCTN achieves the best performance with 4 stacked layers. The model achieves the best performance when the group size is 10, which illustrates that the grouping of variable nodes is beneficial to the model performance, although our original intention of designing the group-range convolution attention is to reduce the time and space complexity of the model. This also verifies that there is no or weak relationship among many variables in the multivariate time series. When the group size continues to increase, more variable information is aggregated and its own characteristics could be ignored, which increases the MAE loss. Figure 8c shows that the increase of model channels will enhance the expressiveness of the model so that the MAE will gradually decrease. However, it will also greatly increase the memory complexity of the model, so we can increase the model channels as much as the memory complexity allows.

## 6. Conclusions

In this paper, we propose a new Transformer-based deep learning model, called STCTN, to improve the multivariate time series forecasting. Within STCTN, local-range convolutional attention and group-range convolutional attention are introduced to solve the difficulties of existing methods in capturing complex spatiotemporal dependencies. Local-range convolutional attention mechanism can simultaneously focus on both global and local context temporal dependencies. Group-range convolutional attention mechanism is designed to model multiple spatial dependency patterns and also reduce the computation and memory complexity. We also proposed continuous positional encoding to link the historical observations and predicted future values in positional encoding and improve prediction performance. Extensive experiments on six real-world datasets show that the proposed method is superior to the existing methods.

In the future, we will apply our proposed framework to other spatiotemporal modeling tasks, such as trajectory prediction. The local-range convolutional attention and group-range convolutional attention can also be used in other sequence modeling and spatial graph modeling tasks respectively, which are left for future work.

## Figures and Tables

**Table 1:** Dataset description and statistics.

	Datasets	#Timesteps	#Nodes	Sample Rate	Start Time	Input Steps	Predict Steps
With priori graph topology	PEMS03	26,208	358	5 min	9 January 2018	12	12
	PEMS04	16,992	307	5 min	1 January 2018	12	12
	PEMS07	28,224	883	5 min	5 January 2017	12	12
	PEMS08	17,856	170	5 min	7 January 2017	12	12
Without priori graph topology	Electricity	26,304	321	1 h	1 January 2012	24	12
	Traffic	17,554	862	1 h	1 January 2015	24	12

**Table 2:** Performance comparison of different approaches on datasets with priori graph topology.

Methods		VAR	FC-LSTM	DCRNN	STGCN	ASTGCN	Graph WaveNet	MTGNN	Transformer	STCTN
Datasets	Metrics									
PEMS03	MAE	23.65	21.16	18.18	17.49	17.69	19.85	17.79	20.01	**16.89**
	MAPE (%)	24.51	23.33	18.91	17.15	19.40	19.31	18.84	23.12	**15.75**
	RMSE	38.26	35.11	30.31	30.12	29.66	32.94	28.75	30.01	**28.02**
PEMS04	MAE	23.75	27.14	24.70	22.70	22.93	25.45	23.31	24.06	**22.53**
	MAPE (%)	18.09	18.20	17.12	**14.59**	16.56	17.29	17.89	17.25	15.21
	RMSE	36.66	41.59	38.12	35.55	35.22	39.70	36.07	37.66	**35.09**
PEMS07	MAE	75.63	29.98	25.30	25.38	28.05	26.85	25.28	28.07	**24.24**
	MAPE (%)	32.22	13.20	11.66	11.08	13.92	12.12	12.48	14.13	**10.12**
	RMSE	115.24	45.94	38.58	38.78	42.57	42.78	38.91	41.42	**37.52**
PEMS08	MAE	23.46	22.20	17.86	18.02	18.61	19.13	17.96	18.93	**17.15**
	MAPE (%)	15.42	14.20	11.45	11.40	13.08	12.68	12.03	13.69	**10.93**
	RMSE	36.33	34.06	27.83	27.83	28.16	31.05	27.76	28.11	**26.63**

**Table 3:** Performance comparison of different approaches on datasets without priori graph topology.

	Models	Horizon 3			Horizon 6			Horizon 12		
		MAE (10^−2^)	MAPE (%)	RMSE (10^−2^)	MAE (10^−2^)	MAPE (%)	RMSE (10^−2^)	MAE (10^−2^)	MAPE (%)	RMSE (10^−2^)
Traffic	VAR	1.63	73.34	3.17	1.84	77.51	3.51	1.95	78.36	3.69
	FC-LSTM	1.68	46.52	4.01	1.71	47.81	4.05	175	52.25	4.02
	Graph WaveNet	1.77	60.49	3.90	1.99	69.08	4.56	1.82	60.56	4.05
	N-BEATS	1.24	38.24	3.00	1.39	49.42	3.23	1.46	44.32	3.40
	MTGNN	1.29	47.28	3.01	1.34	50.82	3.15	1.43	45.46	3.23
	Transformer	1.62	47.53	3.79	1.69	50.56	3.85	1.73	52.03	3.97
	Informer	1.38	43.84	3.38	1.59	45.89	3.42	1.65	47.25	3.59
	**STCTN**	**1.15**	**37.13**	**2.78**	**1.27**	**42.25**	**3.05**	**1.30**	**42.59**	**3.04**
Electricity	VAR	5.96	19.72	8.65	8.58	28.39	12.02	8.97	33.78	13.22
	FC-LSTM	7.03	19.34	9.91	6.99	19.45	9.86	7.16	24.18	10.08
	Graph WaveNet	4.71	14.67	7.22	6.32	21.48	9.17	5.04	16.50	8.08
	N-BEATS	3.41	10.12	5.66	3.73	11.43	6.38	3.90	12.55	7.18
	MTGNN	3.20	10.19	5.23	3.55	11.20	6.11	3.81	12.69	6.55
	Transformer	4.85	15.32	8.43	5.32	17.69	9.25	5.98	19.37	9.91
	Informer	4.05	13.97	7.82	4.45	14.09	8.22	5.07	16.26	8.56
	**STCTN**	**3.09**	**9.15**	**4.85**	**3.43**	**10.52**	**5.43**	**3.68**	**11.61**	**5.85**

**Table 4:** Ablation study on PEMS08 dataset.

Methods	w/o CPE	w/o LCA	w/o GCA	STCTN
MAE	17.61	18.77	18.08	17.15
MAPE (%)	11.47	12.16	12.84	10.93
RMSE	27.57	28.90	28.44	26.63

## Data Availability

Not applicable.
